# Poor Motor Coordination Elicits Altered Lower Limb Biomechanics in Young Football (Soccer) Players: Implications for Injury Prevention through Wearable Sensors

**DOI:** 10.3390/s21134371

**Published:** 2021-06-25

**Authors:** Stefano Di Paolo, Stefano Zaffagnini, Nicola Pizza, Alberto Grassi, Laura Bragonzoni

**Affiliations:** 1 Department for Life Quality Studies, University of Bologna, 40136 Bologna, Italy; stefano.dipaolo@ior.it (S.D.P.); laura.bragonzoni4@unibo.it (L.B.); 2 Department of Biomedical and Neuromotor Sciences, University of Bologna, 40136 Bologna, Italy; 3 Orthopaedic and Traumatologic Clinic II, IRCCS Istituto Ortopedico Rizzoli, 40136 Bologna, Italy; nicola.pizza@ior.it (N.P.); alberto.grassi@ior.it (A.G.)

**Keywords:** wearable sensors, joint kinematics, football, motor coordination, injury prevention, ACL

## Abstract

Motor coordination and lower limb biomechanics are crucial aspects of anterior cruciate ligament (ACL) injury prevention strategies in football. These two aspects have never been assessed together in real scenarios in the young population. The present study aimed to investigate the influence of motor coordination on lower limb biomechanics in young footballers during an on-the-pitch training. Eighteen juvenile football players (10 y ± 2 m) were enrolled. Each player performed a training drill with sport-specific movements (vertical jump, agility ladders, change of direction) and the Harre circuit test (HCT) to evaluate players’ motor coordination. Wearable inertial sensors (MTw Awinda, Xsens) were used to assess lower limb joint angles and accelerations. Based on the results of the HCT, players were divided into poorly coordinated (PC) and well-coordinated (WC) on the basis of the literature benchmark. The PC group showed a stiffer hip biomechanics strategy (up to 40% lower flexion angle, ES = 2.0) and higher internal-external hip rotation and knee valgus (*p* < 0.05). Significant biomechanical limb asymmetries were found only in the PC group for the knee joint (31–39% difference between dominant and non-dominant limb, ES 1.6–2.3). Poor motor coordination elicited altered hip and knee biomechanics during sport-specific dynamic movements. The monitoring of motor coordination and on-field biomechanics might enhance the targeted trainings for ACL injury prevention.

## 1. Introduction

Motor coordination plays a crucial role in injury prevention strategies in football (soccer) at all levels. Such an ability is acquired in the prepubertal period, which is also when children usually approach football, and entails dexterity, neuromotor control, and flexibility. Recent studies [1,2] underlined the importance of training the different aspects of motor coordination already in the youth to reduce the risk of non-contact musculoskeletal injuries, by acting on both intrinsic and extrinsic risk factors. Alongside motor coordination, players’ biomechanics has gained considerable interest in preventative training programs [3,4,5]. Despite their comprehensiveness and quality, these programs did not successfully reduce injury rates in the young population as they did in elite adult players [6,7,8,9], and the incidence of severe injuries (e.g., anterior cruciate ligament, ACL injury) has doubled in the pediatric football players’ population in the last 10 years [10]. Hence, innovative prevention strategies might require a deeper comprehension of players’ biomechanics and motor control interaction.

On-field biomechanical analyses of complex movements through wearable inertial sensor systems are gaining growing interest [11,12,13]. Compared with the in-lab standard assessments, the use of wearables in real scenarios (e.g., sport-specific pitch) extends the opportunities to monitoring players’ motion and offering real-time feedback. These aspects have particular importance for team sports, where the interaction with the environment (teammates, opponents, etc.) is predominant. Nonetheless, on-field multi-joint biomechanics of young football players were investigated in only one study and never in relation to motor coordination. Such an analysis could highlight biomechanical alterations due to poor motor control in the light of injury prevention.

Therefore, the present study aimed to investigate the influence of motor coordination on lower limb biomechanics through wearable inertial sensors in young football players during a typical on-the-pitch training. The hypotheses were that (I) players deemed as having poor motor coordination (dexterity) would elicit altered lower limb biomechanics during football-specific tasks and that (II) these biomechanical alterations would be associated to the risk factors targeted in ACL injury prevention strategies.

## 2. Materials and Methods

### 2.1. Participants

Eighteen juvenile football players (one team) were enrolled in the study. Players were all male, aged 10.4 ± 0.4 years (range 10–11), BMI 18.7 ± 3.8, and had no history of previous musculoskeletal injury. Each player’s parent/tutor signed informed consent and agreed to their own child’s performance data acquisition and treatment for research purposes. The team coach was present and supervised all the data acquisition phases. This study obtained approval from the Bioethical Committee of the University of Bologna (ID: 25861 10 February 2020).

### 2.2. Data Collection

The data collection was performed on the team’s football pitch, equipped with artificial turf. The data were collected in a single day during a standard training at the middle of the regular football season. Every player was asked to perform two different motor activities: (I) the Harre circuit test to evaluate the coordinative motor ability [14], and (II) a training drill with football-specific movements. The former was used to inspect players’ motor coordination, while the latter was used to inspect players’ biomechanics.

The Harre circuit (I) is claimed as one of the most appropriate tests to evaluate prepubertal players’ ability to coordinate complex dynamic movements [15,16,17]. The players were instructed to complete the circuit described by Chiodera et al. [15] (Appendix A) at the maximum speed. In short, the circuit requires the execution of a forward roll and three consecutive passages above and underneath three obstacles [15].

**Figure 1:**
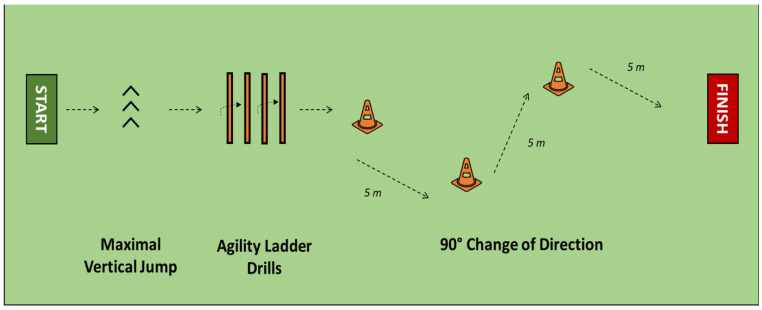
Representation of the football-specific training drill used for the biomechanical assessment. The players were asked to perform the drill at the maximum speed possible. Biomechanical data were recorded through a wearable inertial sensor system.

The football-specific training drill (II) consisted of three tasks (Figure 1): a maximal vertical jump (MVJ); a low skip in the agility ladder drills (ALD); and two changes of direction (COD) at 90° of 5 m each, one right and one left. Such tasks resemble typical movements of football training and matches and have an intrinsic relation with motor coordination: an efficient MVJ requires both muscle explosiveness, intersegmental coordination, and balance during both the push-off phase and the landing phase [18,19,20]; the ALD requires intense and coordinated footwork [21,22]; a 90° COD requires multidirectional acceleration/deceleration phases, and are the basis of the agility tests [23,24]. The players were asked to perform their best and complete the training drill in the lowest time possible. They received only a few indications on how to perform each task to let them move naturally.

Each player performed three unrecorded trials before the real acquisition to become confident with the two activities. The two activities were performed consecutively after a short warm-up.

**Figure 2:**
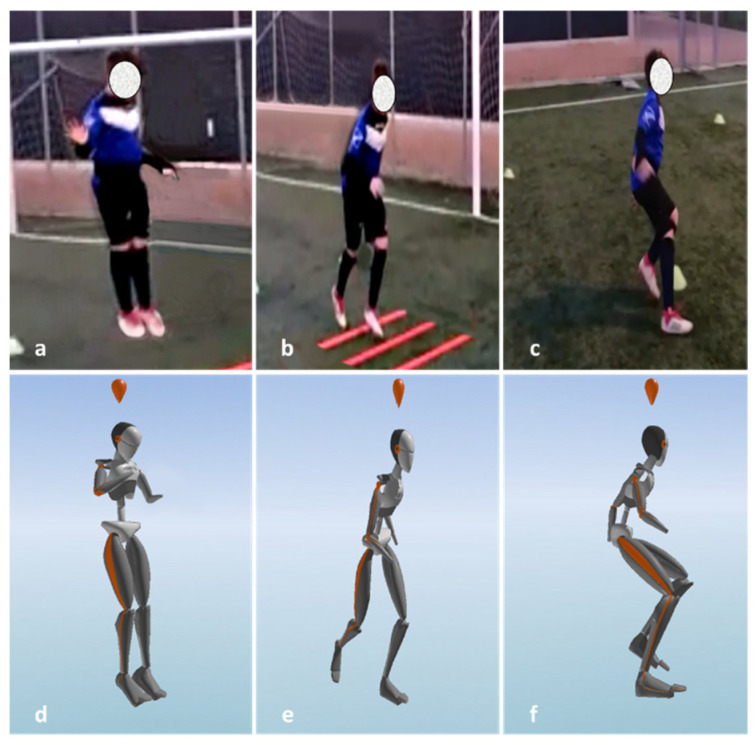
Example of a football player performing the football-specific training drill with the real-time kinematical output from wearables: (**a**,**b**) the maximal vertical jump; (**c**,**d**) the agility ladder drills; (**e**,**f**) the two 90° changes of direction.

Motion data were collected during the football-specific training drill through a set of seven wearable inertial sensors (Xsens MVN, Xsens Technologies, Enschede, The Netherlands) placed bilaterally on the feet, shins, thighs, and lumbar L5 (Figure 2). The sensors (integrated triaxial accelerometers, gyroscopes, and magnetometer) were secured to the body segments through Velcro straps either on the body or on the clothes. A single experienced operator carried out sensor placement for all the players. A static and dynamic system calibration was conducted per single player before the data collection. The accuracy of the wearable sensor system in the kinematical assessment of high-dynamics movements was previously inspected against the gold standard optoelectronic marker-based motion capture [13]: the normalized root mean square error was found to range between 8% and 14% for the sagittal plane joint angles (excellent inter-system correlation) and between 20% and 43% for the frontal and transverse plane joint angles (fair-to-excellent inter-system correlation) in lower limbs and trunk.

### 2.3. Data Analysis

The time elapsed for the Harre circuit test was recorded through a digital soccer watch [15] by a single operator for all the players to the nearest 0.01 s. A benchmark value of 15.7 ± 2.8 s was considered for completing the Harre circuit based on a previous cohort study on 241 healthy children aged 8–10 years [17]. The choice of a literature-based benchmark to inspect players’ coordination was done for the sake of study reproducibility. According to the benchmark value, the players were divided into two groups: the players with time elapsed lower or within the benchmark value ± SD (i.e., <18.5 s) were classified as well coordinated (WC group), while the players with time elapsed higher than the benchmark value ± SD (i.e., >18.5 s) were classified as poorly coordinated (PC group) [25]. In this way, the sub-group of players performing worse than the literature benchmark was split from those performing on average or even better.

Hip, knee, and ankle biomechanics (joint angles and joint accelerations) were recorded for all the tasks of the training drill at a sampling frequency of 100 Hz and HD re-processed for drift inaccuracies reduction. The joint angles on frontal (abduction-adduction, AA for hip and ankle; varus-valgus, VV for the knee), transverse (internal-external rotation, IE), and sagittal (flexion-extension, FE) plane were evaluated in terms of ranges and peak values; the joint accelerations on anteroposterior (AP), mediolateral (ML) and vertical (V) axes were evaluated in terms of ranges, positive peaks (+), and negative peaks (−).

The biomechanical data were evaluated in terms of dominant and non-dominant limbs, respectively defined as the kicking and the standing limb. Furthermore, the limb asymmetry within groups (i.e., the percentage difference between the dominant and non-dominant limb) was computed for each biomechanical variable. Data analysis was performed in a customized Matlab script (The MathWorks, Natick, MA, USA).

### 2.4. Statistical Analysis

The Shapiro–Wilk test was used to verify the normal distribution of the data. Normal-distributed continuous variables were presented as mean and standard deviation (SD), while categorical variables were presented as percentages over the total. The two-way ANOVA was used to assess the statistical differences between the players with coordination (WC and PC groups) and limb asymmetry (dominant and non-dominant limb) as factors. The differences between the single groups were assessed through the two-tailed Student’s t-test with Bonferroni correction for multiple comparisons. The differences were considered statistically significant for *p* < 0.05. Cohen’s d effect size (ES [26]) was calculated alongside *p*-value: ES of 0.2, 0.5, 0.8, 1.4, and 2.0 were considered small, medium, large, very large, and huge, respectively [27]. Only statistically significant differences with ES ≥ 1.5 (very large-huge) were reported in the Results section and further discussed to limit the type II error. All the analyses were performed in Matlab.

## 3. Results

### 3.1. Harre Circuit Test (Coordination)

The WC group players (n = 10) took 16.4 ± 0.9 s to complete the Harre circuit, while the PC group players (n = 8) took 20.5 ± 2.9 s (*p* = 0.009). Demographics (BMI, age, weight, height) were not statistically different between the two groups (*p* > 0.05).

### 3.2. Training Drill (Biomechanics)

A higher peak flexion angle was found in the WC group compared with the PC group for hip joint in dominant and non-dominant limb both in MVJ and ALD tasks (Table 1, Appendix B). In the MVJ task, the WC group showed a higher peak varus angle and a greater range of vertical ankle acceleration than the PC group. In the COD task, the PC group showed a wider range of internal-external hip rotation.

The presence of biomechanical limb asymmetry was found for the PC group players in the COD task in terms of knee varus-valgus range and positive peak and range of vertical knee acceleration (higher values for the dominant limb, Table 2). No limb asymmetries were found in the WC group.

## 4. Discussion

The present study is the first aiming to investigate how motor coordination influences the lower limb biomechanics during on-the-pitch training through wearable inertial sensors in young football players. The most important finding was that biomechanical differences were found between well-coordinated (average and good results in the Harre circuit test [17]) and poorly-coordinated (higher time elapsed in the Harre circuit test [17]) players in all the investigated tasks. In particular, poorly-coordinated players seem more prone to adopt “dangerous” biomechanical strategies in light of non-contact injuries, such as ACL injury [28], thus confirming the hypotheses.

The present study tackles several trend topics in orthopedics, sports medicine, and human movement analysis: the influence of motor coordination on biomechanics towards movement quality and injury prevention; the investigation in a sport-specific real scenario; the focus on a juvenile population, namely one younger than that with the highest odds of non-contact injury and the most severe implications for health and career [3,10,29,30]. The use of wearable sensors for multiple joint biomechanics data collection and processing represents a further significant technological advancement for this type of analysis.

The results suggest that motor coordination influences players’ hip biomechanics. Poorly coordinated players exhibited up to 43% lower hip flexion peak angle than the well-coordinated players in both the MVJ and the ALD tasks regardless of limb dominance. The only previous on-filed multi-joint biomechanical investigation on young football players (11–12 years) through wearable sensors also highlighted differences in hip biomechanics in the drop jump test after a fatigue protocol [31]. A stiff hip biomechanical strategy has been associated with poor core balance and increased intra-articular knee and ankle mechanical loads [32,33,34,35]. The achievement of the sagittal plane range of motion in landings, high-speed movements, and cut maneuvers is a critical target in preventative programs to reduce the risk for lower limb non-contact injury [36,37]. A prospective analysis on 90 adolescent female athletes recently demonstrated a higher relative risk for ACL injury in the presence of limited hip flexion angle [5].

Motor coordination was also associated with frontal and transverse plane biomechanics in the ALD and the COD tasks. The PC group showed higher hip transverse plane rotations, while the WC group showed higher varus peak angles. Both aspects are meaningful to preventative training: e.g., in ACL injury prevention, the main target is the limitation of the external knee moments caused by the coupling of hip internal rotation, hip adduction, and knee valgus [38,39,40,41,42,43]. Hewett et al. [44,45] and Raisanen et al. [46] demonstrated a reduction of injury risk after targeted neuromuscular training focused on frontal plane control in adolescent athletes following ACL-reconstruction. From this perspective, poorly-coordinated players seem to exhibit a more dangerous pattern, while well-coordinated players seem to exhibit a more protective strategy. Since the training drill was performed at the maximum speed possible, i.e., with a performance-driven outcome, it is possible that such patterns also occur during regular training and matches.

Statistically significant differences between dominant and non-dominant limb biomechanics (limb asymmetry) were found only in the poorly-coordinated players. More specifically, differences regarded the knee joint during the COD task, and greater varus-valgus angle and vertical accelerations were found for the dominant limb. This finding supports a connection between poor motor coordination and altered biomechanics: first, the COD is claimed as the most demanding sport-specific task in terms of motor coordination and biomechanics [47] and the most frequent situational pattern for non-contact injuries [28]; second, biomechanical asymmetries are often associated with the risk of muscular and ligamentous non-contact injury [39,48], and are addressed as a pivotal aspect of pre- and re-habilitation; and, third, higher incidence of non-contact injury has been found for dominant limb in football [48,49,50,51]. The presence of limb asymmetries during dynamic movements in the young population might be indicative of sub-optimal neuromotor control development, leading to an increased risk of future injury.

The present study highlighted that poor motor coordination reflects altered biomechanical patterns in young football players. Such patterns are in line with the literature on non-contact ACL injury mechanism and prevention and should be further investigated to offer precious insights into the influence of poor motor coordination and football injuries. In particular, the incidence of dangerous patterns in players with limited coordination might be investigated in the presence of sport-specific external perturbations (e.g., ball, opponents) and after targeted multi-tasking training sessions.

The present study has several limitations. First, the analysis was conducted on a small number of players from a single male football team aged 10–11 years. Thus, the results cannot be conclusive nor generalizable to other sport-specific tasks. Despite the small sample size, the significant differences were all adequately powered (ES ≥ 1.5, power ≥ 80%), thus reducing the risk for type II error. Second, the analysis was performed a single time. Further follow-up acquisitions would have given more robust insights regarding players’ motion variability. Third, the division in well-coordinated and poorly-coordinated players was based on a single parameter (time elapsed in the Harre circuit test) [17]. Although a multiple-test battery could offer a more robust indication of players’ motor coordination [2], the Harre circuit test is a trusted method to assess such an ability in preadolescent footballers [15,17], and has recently been modified to be extended to adolescent ones [52]. The choice of a literature-based benchmark for group split through the Harre circuit test was done to limit the bias related to the analysis of a single cohort of players (one team) in identifying those with poor coordination. Recent studies also proposed the use of joint coordination variability as a predictive measure for non-contact injury risk [53], thus offering a comprehensive investigation of the neuromotor and biomechanical aspects of the movement. Lastly, little knowledge on multi-joint on-field biomechanics is present in the literature, making it difficult to inspect the present study results. In particular, no on-field validation or data collection of football-specific movements is provided. Indeed, the present study represents one of the first attempts to assess football players’ biomechanics on-field during regular training and highlights a possible use of wearable inertial sensors in pediatric injury prevention. Such technology could be a valuable support to coaches and sports physicians in identifying movement patterns and strengthening preventative strategies. The young and competitive population might benefit most from continuous monitoring of coordinative and biomechanical risk factors for ACL non-contact injury.

## 5. Conclusions

Poor motor coordination was associated with altered lower limb biomechanics in young football players during typical movements of training and matches. Biomechanical investigations in real scenarios through wearables with specific regards to motor coordination might enhance the targeted trainings for non-contact ACL injury prevention.

## Figures and Tables

**Table 1:** Biomechanics through wearable sensors assessed according to motor coordination.

	PC Group	WC Group	Diff %	Effect Size	*p*-Value
Joint angles (°)					
Maximal Vertical Jump					
Hip flexion peak, dominant	44.1 ± 7.7	59.8 ± 12.8	−36%	1.5	0.019
Hip flexion peak, non-dominant	42.8 ± 7.9	61.2 ± 10.6	−43%	2.0	0.004
Knee varus peak, non-dominant	−4.8 ± 2.4	−10.3 ± 2.8	−116%	2.1	0.008
Agility Ladder Drills					
Hip flexion peak, dominant	50.4 ± 7.3	64.0 ± 7.8	−27%	1.8	0.006
Hip flexion peak, non-dominant	48.8 ± 8.3	60.2 ± 6.9	−23%	1.5	0.017
Change of direction					
Hip IE range, dominant	28.0 ± 4.6	21.5 ± 4.3	+23%	1.5	0.019
Joint accelerations (m/s^2^)					
Maximal Vertical Jump					
Ankle V range, non-dominant	163.6 ± 31.8	232.3 ± 51.9	−42%	1.6	0.014

Note: Only the significant differences (*p* < 0.05) with at least huge effect size (ES ≥ 1.5) were reported; positive values of diff % mean a higher magnitude for biomechanical variables of PC group; dominant limb means the preferred for kicking. Abbreviations: PC, poorly-coordinated players; WC, well-coordinated players; IE, internal-external; V, vertical.

**Table 2:** Biomechanics asymmetries between dominant and non-dominant limb.

	Dominant	Non-Dominant	Diff %	Effect Size	*p*-Value
Joint angles (°)					
Change of direction					
Knee VV range, PC Group	23.2 ± 6.7	14.5 ± 3.5	+38%	1.6	0.013
Joint accelerations (m/s^2^)					
Change of direction					
Knee V peak (+), PC Group	119.9 ± 19.8	72.7 ± 20.5	+39%	2.3	0.001
Knee V range, PC Group	183.9 ± 36	126.6 ± 37.4	+31%	1.6	0.013

Note: Only the significant differences (*p* < 0.05) with at least huge effect size (ES ≥ 1.5) were reported; positive values of diff % mean a higher magnitude for kinematic variables of the dominant limb; dominant limb means the preferred for kicking. Abbreviations: PC, poorly-coordinated players; VV, varus-valgus; V, vertical.

**Figure A1:**
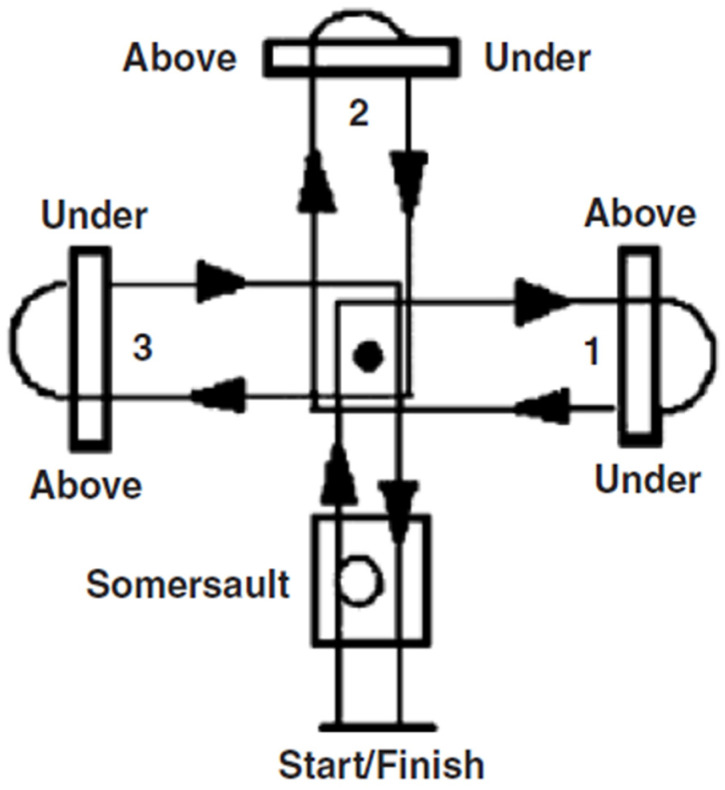
Harre circuit test for the assessment of players’ motor coordination as reported by Chiodera et al. [15].

**Figure A2:**
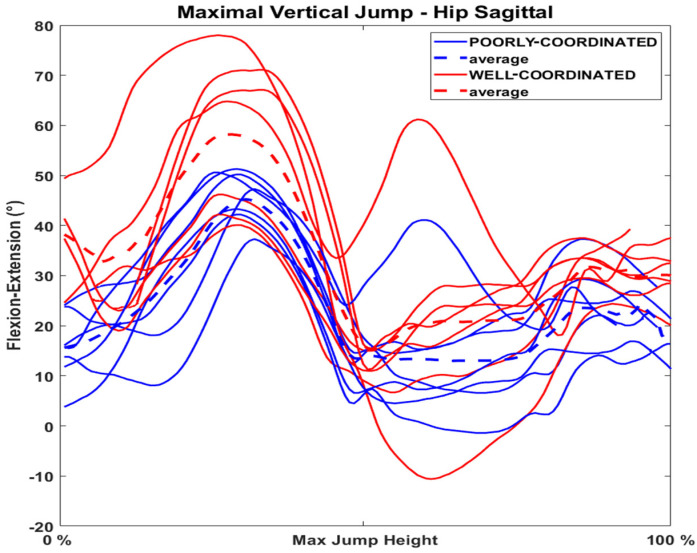
Example of waveform (dominant limb hip flexion-extension during the maximal vertical jump) extracted from the wearable sensors with distinction in poorly coordinated (blue) and well-coordinated (red) players.

## Data Availability

Data are available on reasonable request and due to restrictions, e.g., privacy or ethical.
